# Fumarate drives EMT in renal cancer

**DOI:** 10.1038/cdd.2016.137

**Published:** 2016-11-25

**Authors:** Marco Sciacovelli, Christian Frezza

**Affiliations:** 1 MRC Cancer Unit, University of Cambridge, Hutchison/MRC Research Centre, Box 197, Cambridge Biomedical Campus, Cambridge CB2 0XZ, UK

Fumarate hydratase (FH) is a metabolic enzyme that converts fumarate to malate. *FH* mutations predispose to a highly aggressive and metastatic form of renal cancer. We have recently shown that the accumulation of fumarate in FH-deficient tumour drives an epigenetic reprogramming that contributes to tumour invasiveness.

Cancer is a multifactorial disease thought to be driven by genetic alterations. Mutations in metabolic enzymes of the tricarboxylic acid (TCA) cycle, such as succinate dehydrogenase (SDH), fumarate hydratase (FH) and isocitrate dehydrogenase (IDH), lead to cancer, suggesting that altered metabolism could play an active role in tumorigenesis.^1^ Our laboratory studies how FH loss leads to renal cancer to dissect the link between altered metabolism and cancer.

FH was originally found mutated in hereditary leiomyomatosis and renal cell cancer (HLRCC),^2^ which causes a very aggressive and metastatic type of renal cancer. It has been proposed that fumarate, accumulated upon FH loss, contributes to the molecular features of FH-deficient tumours,^3^ acting as an *oncometabolite.*^1^ For instance, fumarate inhibits prolyl hydroxylases (PHD), *α*-ketoglutarate-dependent dioxygenases (aKGDD) involved in HIF degradation, leading to HIF stabilisation and pseudohypoxia.^4^ Through covalent bonding with reactive thiol residues of Keap1, fumarate was also shown to activate a potent antioxidant response mediated by NFE2-related factor 2 (NRF2).^1^ More recently, it was found that fumarate could also inhibit Ten-Eleven Translocation (TET) proteins, another class of aKGDDs involved in DNA demethylation,^5, 6^ leading to DNA hypermethylation. The role of this epigenetic alteration in tumorigenesis is still unclear. In our recent work,^7^ we have shown that, by virtue of its epigenetic function, fumarate contributes to the invasive features of FH-deficient renal cancers.

Using proteomics and transcriptomics analyses, we demonstrated that epithelial mouse and human FH-deficient renal cells exhibit overlapping mesenchymal features. Further functional characterisation revealed that the loss of FH activates an epithelial-to-mesenchymal transition (EMT), a process involved in both tumour initiation and progression.^8^ EMT is orchestrated by various transcription factors that suppress epithelial genes and activate mesenchymal markers. To investigate how the loss of FH could activate EMT, we measured the expression of several EMT-related transcription factors. We showed that Fh1-deficient cells express high levels of *Zeb1* and *Zeb2* and that miR-200, a family of miRNAs that suppresses Zeb translation, were strongly downregulated in these cells. It was recently shown^9, 10^ that miR-200 expression is regulated by promoter methylation, which, in turn, is controlled by TET demethylating activity. We therefore hypothesised that the epigenetic suppression of miRNA 200 could be caused by fumarate-driven inhibition of TET. In line with this hypothesis, we showed that a regulatory region of *mir200ba429* cluster is hypermethylated in Fh1-deficient cells. Importantly, the epigenetic and phenotypic changes caused by FH loss were recapitulated by the incubation of Fh1-proficent cells with cell-permeable fumarate (see Figure 1 for a schematic of the pathway). These results showed, for the first time, that fumarate triggers EMT via the epigenetic suppression of miR-200.

The link between fumarate accumulation and EMT was also validated in renal cancer samples. First, we found that HLRCC patients exhibited a significant increase in fumarate levels, inhibition of TET activity, and miR-200 suppression compared to adjacent normal tissue. Then, taking advantage of the data from The Genome Cancer Atlas (TGCA), we found that FH loss resulted in hypermethylation and suppression of miR-200 in a larger cohort of FH-deficient renal cancers. Finally, we found that *FH* is downregulated in a set of clear cell renal cell carcinoma, and *FH* expression is positively correlated to EMT markers, confirming the link between FH and EMT *in vivo* in hereditary and sporadic tumours.

Although fumarate accumulation activates a plethora of signalling cascades, our findings suggest that the EMT is a key phenotype of FH-deficient cancer driven caused by fumarate-driven TET-mediated suppression of miR-200. This unexpected property of fumarate is likely shared by other oncometabolites. Indeed, both succinate and 2-hydroxyglutarate, the metabolites accumulated in *SDH* and *IDH* mutant tumours, respectively, are also well-established TET inhibitors. Interestingly, SDH-deficient pheochromocytomas and paragangliomas,^11^ *Sdhb*-deficient cells,^7^ and IDH-mutant cells also exhibit EMT.^12^ Our results indicate that, at least in Sdhb-deficient cells, EMT induction is mediated by epigenetic suppression of miR-200, similar to that observed in FH-deficient cells. These results have important implications. First, they indicate that TET inhibition and EMT induction could be a convergent mechanism of action of these metabolites. Second, they suggest that EMT could be a common response to loss of mitochondrial enzymes and could link mitochondrial dysfunction to tumorigenesis. It is tempting to speculate that environmental conditions that affect mitochondrial function and, as a consequence, the abundance of TCA cycle metabolites, such as ischaemia or hypoxia, could also lead to dysregulation of TET activity, promoting tumorigenesis. In conclusion, our findings go beyond the understanding of how *FH* mutations lead to HLRCC and pave the way for the understanding of how mitochondrial metabolites could promote tumorigenesis.

## Figures and Tables

**Figure 1:**
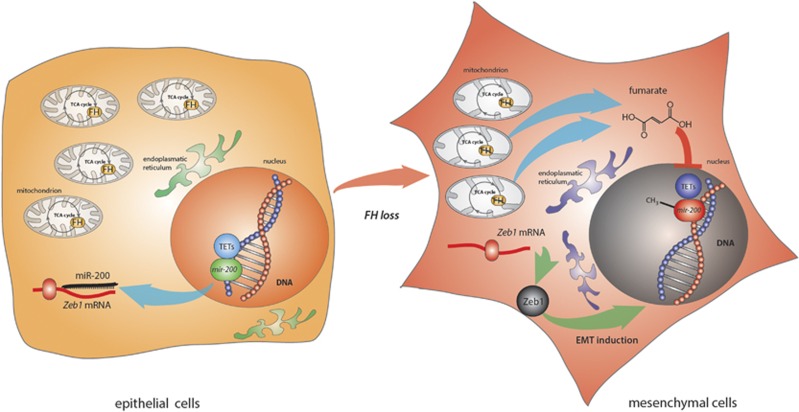
Schematic representation of the proposed link between FH loss and EMT activation. Left panel: In epithelial Fh1-proficient cells, TETs bind the *mir-200* regulatory region, favouring DNA demethylation and miR-200 expression. miR-200 can then bind its mRNA targets such as *Zeb1,* efficiently reducing *Zeb1* translation. Right panel: After FH loss, fumarate is accumulated and inhibits TETs activity, leading to *mir-200* DNA hypermethylation. The reduced expression of miR-200 favours *Zeb1* translation and subsequent EMT reprogramming. Cells switch their phenotype, moving from an epithelial to a mesenchymal configuration
